# Drying soil in North China drove the outbreak range expansion of meadow moth by facilitating long-distance migration

**DOI:** 10.1038/srep30370

**Published:** 2016-07-25

**Authors:** Xiao Chen, Yuying Jiang, Aiguo Kang, Baoping Zhai

**Affiliations:** 1 Department of Entomology, College of Plant Protection, Nanjing Agricultural University, Nanjing 210095, China; 2 National Agro-Tech Extension and Service Center, Beijing 100125, China; 3 Plant Protection Station of Kangbao, Kangbao 076650, China

## Abstract

Studies of the mechanism underlying the range expansion of organisms have mainly focused on environmental conditions at the edges of species’ distributions, potentially ignoring other possible factors. Here, we demonstrated the outbreak range expansion of meadow moth from North China to Northeast China in the past three outbreak periods. We found that the negligible infestation in Northeast China in the 1950s could not be explained by local climatic conditions. However, the soil moisture in North China decreased distinctly from 1951 to 2011 and was significantly and positively correlated with the timing of the first adult peak on plateaus, meaning that the deterioration of habitat conditions could result in earlier peaks of adults in areas of high-elevation by stimulating the short-distance dispersal of adults from the plains to the plateaus. Adults peaking earlier have a stronger tendency to emigrate due to mismatched phenology. Hence, drying soil in North China caused the frequent long-distance migration of meadow moth after the 1970s and drove the outbreak range expansion. This study suggests that, for a migratory species, the deterioration of habitat conditions in overwintering areas might also influence the distribution of this species in breeding areas at high latitudes by facilitating migration activities.

The range limits of species have long been a central theme in ecology. Range expansion towards higher latitudes and elevations has been recorded in many taxa^1^,^2^,^3^,^4^,^5^,^6^. To date, studies of the mechanism underlying range expansion have mainly focused on the edges of species’ distributions, with climatic variables, species interactions and habitat conditions being the main factors considered^7^,^8^,^9^,^10^,^11^,^12^, potentially ignoring other possible factors. Range expansion might also be affected by long-distance dispersal/migration. For example, the long-distance dispersal of mountain pine beetle plays an important role in the early stage of the current outbreak in British Columbia, Canada^13^. Further more, migration activity could be influenced by the climate pattern in wintering quarters, as reported for some species of trans-Saharan migrant birds^14^. Land degradation in dry grassland is increasingly serious due to climatic variations and human activities, which might influence the migration activity of a migratory species that overwinters in grassland and thus may affect its breeding performance at the northern margin of its distribution. However, no studies have addressed this issue.

Here, we analysed the causation of the outbreak range expansion of meadow moth in China in the past sixty years and showed that drying soil in North China drove the outbreak range expansion of meadow moth towards Northeast China by facilitating its long-distance migration.

Meadow moth (*Loxostege sticticalis* L.) is a destructive insect pest inhabiting drylands in the northern temperate zone^15^,^16^,^17^,^18^. It infests more than 200 species of plants and can cause large economic losses to agricultural and pastoral production during eruptive outbreaks. Abundant larvae can eat leaves and tender stems of host crops, such as sugar beet, beans, sunflower, pea, maize, and vegetable plants, and can lead to the death of entire plants. In China, the primary overwintering area of meadow moth is in the agro-pasture interlacing zone in North China^19^, where severe land degradation has occurred over the past several decades^20^.

Meadow moth exhibits intermittent population dynamics. Three outbreak periods, namely, 1951–1960, 1977–1984, and 1995–2009, have been recorded in China over the past 60 years^19^,^21^,^22^,^23^. Historical records have revealed that the outbreak range of meadow moth differs distinctly in various periods. Meadow moth infestations were limited mainly to North China in the first period, but crossed the East Asia Desert and expanded to Northeast China in the next two periods, with Songnen Plain (including the midwestern part of Heilongjiang Province and the northwestern part of Jilin Province) becoming the most severely damaged area (Fig. 1).

Climatic conditions have profound effects on meadow moth infestations. Because the occurrence of meadow moth crosses different climatic zones from desert steppe to forest steppe, the limiting factors for mass breeding are quite different. The southern distribution limit of meadow moth lies in the arid steppe where humidity is the most critical factor for mass breeding. In contrast, the northern distribution limit of meadow moth lies in steppe-forest districts, where the climate is sufficiently humid but not so warm making temperature the most critical factor for mass breeding^24^. The low temperatures in spring could limit population increase or even lead to population collapse. For example, overwintered adults in Heilongjiang Province in 1983 were rather abundant; however, the density of first-generation larvae was very low because of abnormally cold weather in the spring^25^. Consistent with global warming, the average temperature in Northeast China has increased markedly since the 1980s^26^. Hence, a lower temperature in the 1950s may be a possible cause of the negligible infestation.

Meadow moth infestations in Northeast China also depend on the long-distance migration of adults from the primary overwintering area in North China^27^,^28^, especially before the 1990s, when meadow moth seldom overwintered locally. Currently, such long-distance northward migration seems to be a very common phenomenon. The topography in the primary overwintering area is complex, with Ulanqab and Bashang plateaus located in the north and mountains and valleys interlacing in the south. On the plateaus, local cocoons usually emerge around 5–10 June (unpublished data, observed by one of the authors, Kang A.G.). However, a large number of moths can often be observed by the end of May^23^. We could reasonably infer that these moths come from low-elevation areas in the south or the west, where overwintered adults emerge much earlier than do those on the plateaus due to higher temperatures. At that time, habitat conditions on the plateaus are still very adverse for meadow moth breeding: the temperature is low, and there are no flowering plants^29^. These immigrants would emigrate massively once the southerly wind blows and the air temperature on the ground increases to above 20 °C. After flying for a whole night, a moth swarm could arrive at the East Asia Desert, where harsh environmental conditions would stimulate the moths to continue their northward migration the following night. Usually, a moth swarm can reach Songnen Plain after two or three nights of flying by means of a low-level jet before a cyclone. Therefore, the short-distance upslope dispersals of overwintered adults may be the key factor influencing their long-distance migration. The vegetation conditions in grassland in the 1950s were much better than those at present and thus might be favourable for overwintered adults in the plains to breed locally, decrease their short-distance upslope dispersal, and thus reduce the frequency of mass migration to Northeast China.

Because no monitoring of meadow moth adults was conducted in Northeast China before 1979, we are not sure whether mass northward migration in the 1950s occurred as frequently as it currently does. Hence, two hypotheses were tested to explore the causes of the outbreak range expansion of meadow moth: (1) the climatic conditions in Northeast China in the 1950s were very adverse and could not support the breeding of immigrant adults; and (2) the habitat conditions in North China in the 1950s were very favourable so that overwintered adults in the plains seldom migrated to the plateaus, making mass northward migration rare.

## Results

### Influence of climatic conditions in Songnen Plain on the infestation of meadow moth

**Figure 2:**
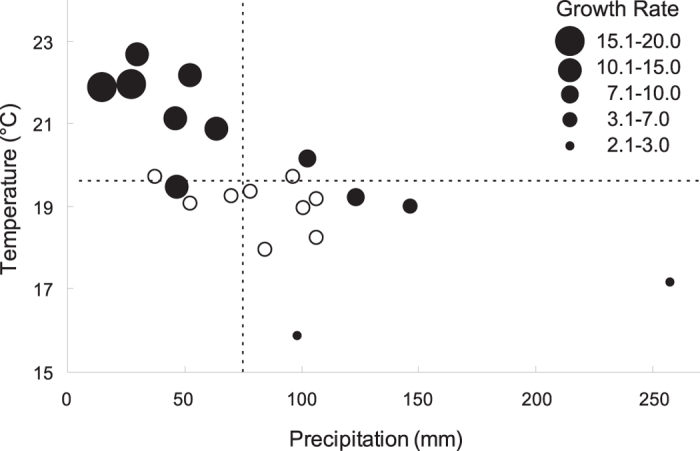
Scatter plot of the average temperature and precipitation in June across five meteorological stations in Songnen Plain (the distribution of stations is shown in Fig. 6) in the first outbreak period (open circles) and the next two periods (solid circles of varying size). The varying size of the solid circles indicates the population growth rate after 1981 from the overwintered adults to the first-generation larvae as calculated from the monitoring data. Dashed lines show the long-term mean of the average temperature and precipitation from 1961 to 1990.

A warm and dry climate is favourable for the rapid population growth of meadow moth in Songnen Plain (Fig. 2). The moth population was able gain a high growth rate when the mean temperature was greater than 20 °C and when precipitation was less than its long-term mean. When the temperature was close to its long-term mean, population growth rates were distinctly mediated by precipitation. A higher precipitation corresponded to a lower growth rate, and population was restrained when precipitation was greater than 130 mm. When temperature was less than 18 °C or when precipitation was greater than 200 mm, population collapse would occur.

Favourable climatic conditions occurred in 7 of the 9 years in the first period (77.78%) and in 18 of the 21 years in the next two periods (85.71%). The frequency of favourable years did not differ significantly between the different stages (Fisher’s test, p = 0.622). Meadow moth infestation occurred in 1 of the 7 favourable years in the first period (14.29%) and in 12 of the 18 favourable years in the following two periods (66.67%). The difference in the infecting frequency in favourable years was significant between stages (Fisher’s test, p = 0.03021). This result indicates that some factors other than local climatic conditions strongly affect the meadow moth infestation.

Therefore, the negligible meadow moth infestation in Songnen Plain in the 1950s cannot be explained by local climate conditions.

### Long-term trends in soil moisture and emergence date of adults in the primary overwintering area

**Figure 3:**
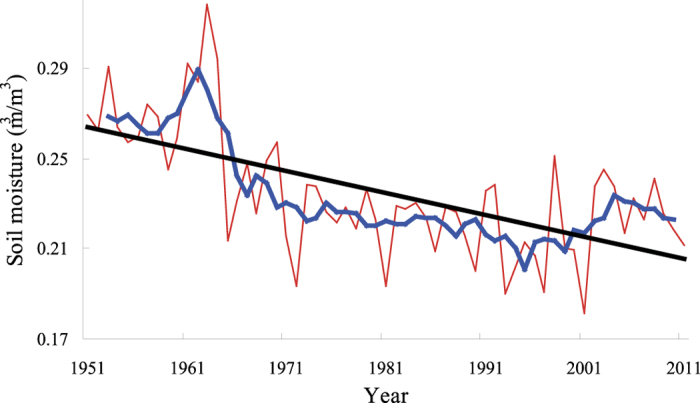
Variation in the mean soil moisture in May and June in the primary overwintering area of meadow moth (red curve) and their 5-year moving average (blue curve). The long-term trend is indicated by a straight line.

The soil moisture in May and June in the primary overwintering area decreased significantly from 1951 to 2012 (R^2^ = 0.3755, d.f. = 60, p < 0.001, Fig. 3). These data indicate that the vegetation conditions were rather good before the early 1960s but severely deteriorated after 1965.

**Figure 4:**
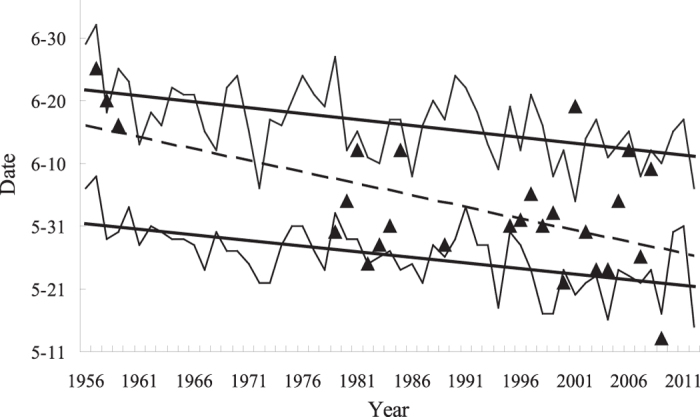
Theoretical dates of emergence of meadow moth in the plains (lower curve) and on the plateau (higher curve), observed dates of the first peak flight of meadow moth on the plateau (triangles) and their long-term trends (straight lines).

The theoretical dates of adult emergence in the plains advanced by approximately 1.72 d/10 yr during the past several decades (R^2^ = 0.318, d.f. = 55, p < 0.001, Fig. 4). The theoretical dates of emergence on the plateaus showed a very similar trend. The observed dates of the first adult peak on the plateaus mainly fluctuated within these two curves (Fig. 4) but advanced faster than did the theoretical dates of adult emergence. The observed dates from 1956–1958 were all near the dates of emergence on the plateaus, suggesting a local source. However, after 1979, the first adult peak on the plateaus often occurred much earlier than did local emergence, indicating frequent dispersals from areas of lower-elevation.

### Relationship between soil moisture and the relative timings of the first adult peak on the plateau

**Figure 5:**
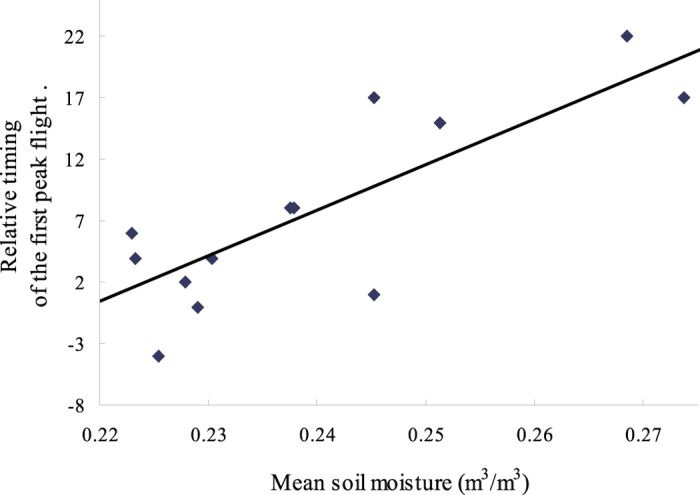
Significant relationship between the relative timing of the first peak flight of meadow moth on the plateau and the mean soil moisture in May and June.

The relative timing of the first adult peak on the plateaus (defined as the number of days for which the observed date of the first adult peak on the plateaus lagged behind the theoretical date of adult emergence in the plains) was significantly and positively correlated with the soil moisture (r = 0.824, d.f. = 12, p < 0.001, Fig. 5). That is to say, the first adult peak on the plateau occurred earlier when soil moisture was lower, indicating that drying soil could facilitate short-distance migration from the plains to the plateaus.

## Discussion

Although the climatic conditions in Songnen Plain in the 1950s were colder and wetter than they are now, the frequency of favourable years for meadow moth is not significantly different. The negligible infestation of meadow moth could not be attributed to local adverse climatic conditions. Soil moisture in the primary overwintering area decreased significantly from 1951 to 2011 and was significantly and positively correlated with the relative timing of the first adult peak on the plateaus. This result shows that the deterioration of habitat conditions could cause earlier peaks of adults in high-elevation areas by stimulating the short-distance dispersal of adults from the plains to the plateaus. Adults peaking earlier have a stronger tendency to emigrate due to mismatched phenology. Hence, drying soil in North China resulted in frequent long-distance migrations of meadow moth after the 1970s and, as a result, drove the outbreak range expansion. Previous studies on latitudinal range expansion have usually focused on the changes in biotic or abiotic factors in the northern margin of distribution. Our study suggests that, for a migratory species, the deterioration of habitat conditions in the overwintering area may also influence its distribution at high latitudes by affecting migration activities.

A drying trend in soil is a reflection of desertification. Desertification is considered a major ecological and environmental threat that directly affects food safety, health, material needs, and other aspects of the livelihood of 250–300 million people^30^. Desertification has important impacts not only at the local scale but also at the regional and global scales^31^; it could modify climate, global biogeochemical cycles, and human geography. However, the regional influence of desertification on the biosphere has not been realised. In our study, desertification in the agro-pasture interlacing zone in North China facilitated the frequent emigration of meadow moth and made Songnen Plain the most severely damaged area. Songnen Plain is approximately 1000 km from the primary overwintering area. The frequent outbreaks in this region caused serious economic consequences, as well as ecological and environmental problems due to large pesticide applications. Hence, our study also provides evidence of the regional influence of desertification on the biosphere and implies that the actual threats of desertification to humans may be more serious than previously suspected.

Long-distance insect migration would bring additional benefits to migrants in addition to escaping deteriorating conditions^32^. The exploitation of the seasonal breeding resources of migrants at high latitudes will result in a greater reproductive productivity in migrants than in non-migrants. Migration activity may also help meadow moth to escape from its natural enemies. As a result, the total area damaged by meadow moth in China in the second and third periods was far larger than that in the first period^33^.

To determine the causation of the long-term change in migration activity, we focused on the short-distance dispersal of overwintered adults from the plains to the plateaus. This process causes long-distance northward migration from the plateaus and seems to be affected by habitat conditions. Although this mechanism is important, two other mechanisms cannot be ignored. First, the desertification of grasslands may directly increase the migration tendency of the adults emerging locally on the plateaus. Second, the advanced emergence of adults may lead to a deviation of adult flight to the annual green-up of grasslands and stimulate frequent emigrations. Over the past 60 years, the emergence of adults has advanced by approximately 10 days (Fig. 4); further advances could be expected in the future because of climate warming. The occurrence of the rainy season in the primary overwintering area has also advanced in a similar trend (data not shown). If advances in the rainy season cannot keep up with those of adult emergence in the future, more frequent migrations could be expected due to mismatched phenology. These mechanisms warrant further studies.

In addition to long-distance migration, the local overwintering pattern also influences the infestation of meadow moth in Songnen Plain. Overwintering cocoons were rarely observed in Northeast China in the 1980s^25^,^34^,^35^. With climate warming, a high density of overwintering cocoons has been frequently found since the 1990s^24^,^36^,^37^. We also estimated an increase in the overwintering frequency in Northeast Mongolia through a source analysis of immigrants^38^,^39^. As a result, migrants from North China could only account for one-third of the immigration peaks in Songnen Plain from 1997 to 2007, and the steppe zone north of the East Asia Desert has become a major source area of overwintered adults^38^. The expansion in source area greatly exacerbated the infestation of meadow moth in Songnen Plain in the third period^36^. However, the steppe zone north of the East Asia Desert could only provide a suitable overwintering area in some years and could not act as a “permanent breeding focus”. The annual northward migration of the meadow moth from North China to Northeast China still plays a decisive role in sustaining its infestation at high latitudes.

## Methods

### Moth Monitoring

Meadow moths in China were routinely monitored by the plant protection station in each county. The abundance of adult meadow moth was monitored with light trapping and field surveys. Light trapping was carried out using a black-light trap with a 20 W light bulb, and catches were counted daily. Field surveys were performed every day from 09:00 to 10:00 local standard time (LST, UTC + 8 h) in the adult flight period. Two or three transects were chosen from each type of vegetation, including crop, grassland, and woodland. A surveyor walked one hundred steps in a normal stride in each transect and visually counted the number of moths being disturbed. The abundance of larvae was investigated when they appeared in large numbers. Three plots were chosen from the main host crop, and each plot contained five sites. An area of 33.3 cm × 33.3 cm was surveyed at each site. Historical monitoring data from each station were collected and provided by the National Agricultural Technology Extension and Service Center of China.

### Regional population growth rate in Songnen Plain

**Figure 6:**
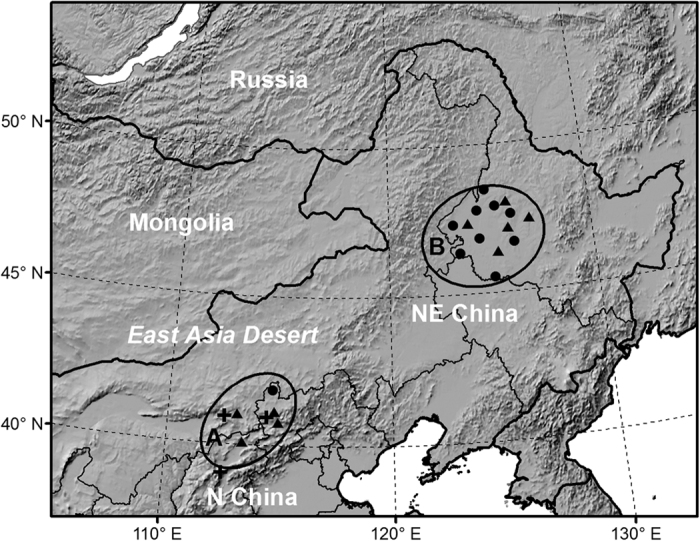
Location of the study sites and meteorological stations. *Oval A* indicates the primary overwintering area of meadow moth in China. *Oval B* represents the most severely damaged area of meadow moth in China, mainly located within Songnen Plain. *Circles* denote the routine monitoring stations of meadow moth. *Triangles* are the meteorological stations. *Crosses* indicate the grids of soil moisture data used in this study. The topography is indicated by shading. This map was created by ArcMap 9.3 (http://www.esri.com).

The regional population growth rate of meadow moth from overwintered adults to the first-generation larvae in Songnen Plain was calculated every year in the second and third periods. First, the peak value in the number of overwintered adults in the field and the following peak value in the density of the first-generation larvae were extracted for each station and each year from the original monitoring record. Then, nine monitoring stations with relatively complete historical records were selected (Fig. 6). The number of adults (individuals disturbed by one step) and the density of the larvae (individuals per square metre) were summed across these nine stations. The ratio of the density of the larvae to the number of adults was used to approximate the regional population growth rate of meadow moth. There were a total of 23 years in the second and third periods, namely, 1977–1984 and 1995–2009, respectively. Considering that the routine observation of meadow moth in most stations in Songnen Plain began in 1982 or 1983, we excluded 1977–1981 from our analysis owing to scarce records. We also precluded 1984, 1995, 2000, 2005, 2006, and 2008 because there were very few overwintered adults in these years; thus, the following first-generation larvae were scarce or even not observed at all. Finally, we obtained the growth rate in 12 years: 1982–1983, 1996–1999, 2001–2004, 2007, and 2009.

The monthly mean temperature and precipitation from five meteorological stations in Songnen Plain were used in this study (Fig. 6): Qiqihaer (47.38°N, 123.92°E); Hailun (47.43°N, 126.97°E); Anda (46.38°N, 125.32°E); Keshan (48.05°N, 125.88°E); and Mingshui (47.17°N, 125.9°E). Data were obtained from the China Meteorological Data Sharing Service System and averaged every June to represent regional climatic conditions.

### Proxy of habitat conditions in the primary overwintering area

Normalized Difference Vegetation Index (NDVI) was widely used in studies on variation in vegetation cover. However, NDVI was unavailable before the 1970s. Because humidity is a key limiting factor of organisms in dry grassland, the monthly mean volumetric soil moisture (0–10 cm) provided by the National Center for Environmental Prediction was used in this study as a proxy of habitat conditions. Three grid points were located within the primary overwintering area (Fig. 6). The average value at these three grid points in May and June were calculated from 1951 to 2009.

### Proxy of mass emigrations on the plateau

Migration is an adaptation to seasonal and regional variation in habitats. In late May and early June, habitat conditions on the plateaus in the primary overwintering area are very adverse; hence, immigrant adults would migrate again when possible. When local adults on the plateaus emerge around 10 June, the temperature increases distinctly, and some plants begin to flower. Meadow moth in this period can breed locally if they do not encounter severe drought or cold snaps. The habitat conditions on the plateau usually become very favourable in the second half of June. Adults flying in this period always remain to breed. Hence, the emigration tendency of meadow moth on the plateaus decreases as the season progresses, and the timing of the first peak on the plateaus is indicative of massive emigration. To remove the influence of the annual fluctuation in temperature, we defined the relative timing of the first adult peak as number of days for which the observed date of the first adult peak on the plateaus lagged behind the theoretical date of adult emergence in the plains.

The observed date of the first adult peak was defined as the first day when more than 300 individuals were caught in a black-light trap. The dates after 1979 were obtained from the light-trap catch records at Kangbao Station (114.60°E, 41.87°N, Fig. 6). Kangbao is located on Bashang Plateau and is the most important monitoring station of meadow moth in the primary overwintering area. The observed dates from 1957 to 1959 were obtained from records of light-trap catches at Zhangbei Station (114.70°E, 41.15°N)^20^. Zhangbei is 80 km south of Kangbao and has a similar physical geography. We obtained records for a total of 24 years: 1957–1959, 1979–1984 and 1995–2009. Of these 24 years, scarce or no overwintering cocoons (density of overwintering cocoons <1/m^2^) were found in the plains in the following 10 years: 1979, 1981, 1995–1997, 1999, 2001, 2005–2006, and 2008. These years were excluded from our analysis.

Insects are ectotherms, and their developmental duration is mainly driven by temperature. The annual theoretical dates of emergence were calculated for the plateaus and for the plains based on the daily mean temperature that was recorded at four meteorological stations (Fig. 6). The average temperatures in Zhangjiakou (40.78°N, 114.88°E, 724.2 m.a.s.l.), Weixian (39.83°N, 114.57°E, 909.5 m.a.s.l.) and Datong (40.1°N, 113.33° E, 1067.2 m.a.s.l.) were used to calculate the theoretical dates of emergence on the plains. The average temperatures in Jining (41.03°N, 113.07°E, 1419.3 m.a.s.l.) and Zhangbei (114.70°E, 41.15°N, 1393.3 m.a.s.l.) were used to calculate the theoretical dates of emergence on the plateaus. The threshold temperature and the thermal constant of meadow moth pupae were 11.6 °C and 158.3 day-degrees, respectively^16^. The daily mean temperature was obtained from the China Meteorological Data Sharing Service System.

### Data Analysis

To test the first hypothesis that the negligible infestation of meadow moth in Songnen Plain in the 1950s was caused by adverse climatic conditions, we first determined the threshold values of the climatic conditions that were favourable for meadow moth infestation by comparing average temperatures, precipitation, and population growth rates since 1982. Then, these threshold values were used to identify the favourable years in the 1950s. Finally, Fisher’s test was performed to determine the significant differences in (1) the frequency of favourable years and (2) the frequency of infestations in favourable years among different stages. The first year of each outbreak period was not included in the analysis, because the meadow moth population was still in the recovery stage at this time and could not migrate to Northeast China en masse. Hence, the scope of comparison was 1952–1960 for the first period and 1978–1984 and 1996–2009 for the next two periods. Because three meteorological stations in this study began observations in 1953, only two meteorological stations were available in 1952 somewhat decreasing the representation of climatic data in this year.

To test the second hypothesis that overwintered adults in the plains seldom migrated to the plateaus due to favourable habitat conditions and rarely migrated northward en masse, we analysed the trends in soil moisture (as a proxy of habitat conditions) in the primary overwintering area using simple linear regression and explored its relationship with the relative timing of the first adult peak on the plateau (as a proxy of massive emigration).

## Additional Information

**How to cite this article**: Chen, X. *et al*. Drying soil in North China drove the outbreak range expansion of meadow moth by facilitating long-distance migration. *Sci. Rep.* **6**, 30370; doi: 10.1038/srep30370 (2016).

## Figures and Tables

**Figure 1:**
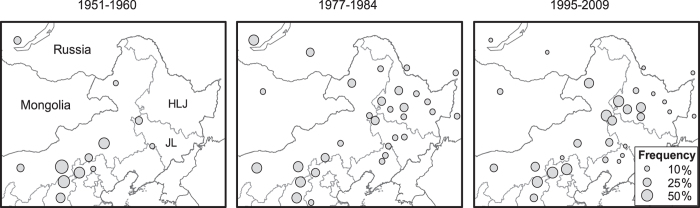
Severe damage frequency caused by meadow moth in Northeast Asia in the three outbreak periods. *HLJ* represents Heilongjiang Province and *JL* represents Jilin Province. This map was created by ArcMap 9.3 (http://www.esri.com).
